# Some Experience Gleaned from Eye and Ear Practice

**Published:** 1898-01

**Authors:** E. C. Ellett

**Affiliations:** Memphis, Tenn.


### SOME EXPERIENCE GLEANED FROM EYE AND EAR PRACTICE.

By E. C. ELLETT, M.D.,
MEMPHIS, TENN.

When I gave the title of my contribution to this meeting to the secretary, I thought to touch on several subjects of every-day interest in practice, and tell some of the conclusions concerning them to which such observation as I have had the opportunity of making has led me. But the first two or three subjects which came to my mind suggested so much to say, that I have been compelled to limit my number of subjects to a very few, and while I realize that I must be brief, yet I am equally alive to the fact that I can do my subject an injustice by being too brief. I want to say that I present only my own opinions, and that I do not lay claim to much originality. I hope to make my position tenable, without being offensively dogmatic.

With this, by way of introduction, I will open my subject proper by a very brief allusion to a topic which is well-worn, but none too well-worn,

### PURULENT OPHTHALMIA OF THE NEW-BORN, the so-called ophthalmia neonatorum. Since I addressed you three years ago, at some length on this subject, my opinions have not changed. In the first place this disease ought not to occur. If it were in the power of the medical profession to throttle yellow fever as effectually as it is in their power to throttle this disease, this Sunny South would not have been invaded with pestilence three months ago. It is just as much our duty to keep the babies from going blind from this disease, as it is to try to keep the babies' fathers and mothers from dying of yellow fever. Clean obstetrics and a drop of two per cent. nitrate of silver solution will do it. I will not quote you any statistics from far-off Germany to prove this, but my own experience in the City Hospital here. About three years ago I was asked to see at that institution, in a very short space of time, several babies suffering with this disease, and at my suggestion the nurse and internes adopted the Credé method to which I alluded, that is, a drop of two per cent. nitrate of silver dropped in each eye after birth. The disease was banished. Some months later it reappeared, and after seeing several cases I inquired into the matter and found that it had been ordered to stop the prophylactic measure. I had it resumed, and for nearly two years I have not seen anything there worse than an evanescent catarrhal conjunctivitis. Therefore I say from my own personal experience that this method of prevention is effective, for the large majority of the women delivered in that institution have a specific vaginitis.

If the disease does develop it is necessary to keep the eyes washed out with a boracic acid solution every half hour or hour, according to the amount of discharge, and the everted lids should be painted once a day with a two per cent. solution of nitrate of silver. This is quite a well tried plan of treatment and leaves little to be desired.

A new remedy with which I have been favorably impressed in this disease is argonin. I have said in detail what I have to say of this drug in the November number of the Memphis *Medical Monthly*, and I will only say here that I would use a five per cent. solution of argonin instead of nitrate of silver in any case which I could see twice a day. You will of course understand that this is a mere outline of the treatment, and I do not try to mention the numerous remedies which may be used. There are other good lines of treatment, which may be known to many of you, but what I have mentioned is the simplest of all, and as efficient as any.

Another subject which I consider very important and on which I have some very decided opinions, is the

USE OF A MYDRIATIC FOR TESTING REFRACTION

in persons under forty years of age. I might profitably consume all my time in a discussion of this subject, so great is its importance, but I will try to be brief.

I use the word mydriatic or "pupil dilator" in deference to custom: a cycloplegic or "accommodation paralyzer" is what I really mean, since the effect we seek with these drugs is paralysis of accommodation. The effect of these substances, of which atropia is the type, is as you know, by paralyzing the focusing apparatus, to render vision indistinct, and indeed impossible for near objects for a length of time which varies from two days with homatropine to ten days with atropia. It is argued against the use of a mydriatic.

1. It is unnecessary.
2. It takes too much time.
3. It may produce toxic symptoms or glaucoma.

In reply to these objections I will say:

1. It is necessary. Unless we resort to this aid, we have only the ophthalmoscope to stand between us and the opticians, and we cannot expect much better results than they obtain. Refracting young people without a mydriatic is work in the dark, and is about as scientific as a physical examination of a person with all his clothes on, or examination of urine by ocular inspection alone. In my own practice I have had innumerable demonstrations of the value of the mydriatic, and the more cases I see the more I am convinced of its utility. I will mention only a few recent cases which illustrate some of the benefits.

A lady came to me last summer who had been given up by an oculist as a case that glasses would not relieve. Under a mydriatic (which he had never even suggested) she was fitted at once with glasses which she now tells me are an "indispensable nuisance," that is, as much as she dislikes to wear glasses, she cannot be comfortable without them. Another case of mixed astigmatism was sadly missed by a gentleman who does not favor the use of mydriatics, and from the same hands I saw a case with near-sighted astigmatism in one eye and far-sighted astigmatism in the other, easily discovered with the accommodation paralyzed, which, to judge by her former glasses, was not suspected without it. This lady I may add, has become one of my most valuable friends, so much has she been relieved by proper glasses.

While writing this two young ladies came to me wearing strong near-sighted astigmatic lenses, fitted in one case by an unbeliever (an oculist) and in the other by a "refracting optician," when the trouble really was exactly the opposite condition, that is, far-sighted astigmatism, in each case. Four years ago, in a paper on "Spasm of the Accommodation," read before this society, I pointed out how this state of affairs is brought about, and in these cases it was impossible without paralyzing the ciliary muscle to arrive at a correct diagnosis.

2. They say it takes too much time. Who consumed most of my first patient's time, the one who had her wearing several pair of glasses, and coming often to him for changes, or the one who took, it is true, a couple of hours for his first examination, but has seen her only twice since in three months? Then, people come to be relieved. They come often with reflex troubles, and by using homatropine I can do conscientious work and keep them from their business but half a day. Is that too much time to take to relieve a man of a headache which, maybe, incapacitates him from business two days in a week, or an eye pain that so destroys his energy as to stand between him and success? My own experience tells me that the days spent under the influence of a mydriatic were profitably though idly spent. If it is absolutely necessary for the patient to continue with the use of his eyes, he can do so, for you have only made him temporarily presbyopic, and a strong convex lens will enable him to read. I keep several such glasses to lend to my patients, and in addition I usually put a drop of eserine in the eye, which hastens the recovery of the ciliary muscle. I prefer, however, that they do not use their eyes, but get the benefit of absolute rest for the two days.

3. Any mydriatic may produce toxic symptoms of glaucoma. Yes, it may. I have seen toxic symptoms occasionally from atropia, but I very rarely use atropia for refraction. In a very considerable number of cases I have seen toxic symptoms twice from homatropine, but not alarming in either case. I have seen symptoms more often than that from cocain, but we do not, on that account stop using cocain. Occasional deaths occur from ether and chloroform, but that does not banish them from our armamentarium. Lately I have been using a combination of homatropine and cocain, one-fiftieth of a grain each, in gelatin disks, and these are slowly absorbed when put in the eye, quite effective, and so far I have noticed no unpleasant symptoms. The danger of causing glaucoma is very small, particularly if you instil a drop of eserine solution (a quarter of a grain to the ounce) in each eye after completing the test, and do not use a mydriatic in cases which present suspicious symptoms.

As additional reasons for the use of the mydriatic, I want to say that the "shadow test," the only objective test of refraction that is of any value, is impossible with an undilated pupil; that eye-strain is composed of two parts, strain of the ciliary muscle and strain of the extra ocular muscles, and the association between these two is so intimate that we can only separate them and study each by itself after the suspension of the function of the ciliary muscle by a mydriatic; that work done without mydriasis is, as I mentioned above, not far removed, in method or result, from the work of the optician, and it behooves us to use this ready means at our command to convince a somewhat reluctant public that there is as much difference between oculists and opticians as there is between physicians and counter-prescribing druggists.

But, without retreating in the least from this position, I must say that cases present themselves which, for a variety of reasons, we may be compelled to test without a mydriatic. In some of these a very satisfactory result is obtained, especially in skillful hands. But no matter how skillful he is who works in this way, it is but a step beyond guesswork. A guess that "hits" is all right, but they will "miss" oftener than they "hit." When I am compelled to prescribe under these circumstances, I do it with the understanding that a mydriatic test may be necessary later.

STRABISMUS, or "crossed eyes," is a subject concerning which we have much to learn. I have convinced myself that in young children it can usually be cured by glasses. In older children, that is, over twelve years of age, and adults, where the squinting eye has become almost or quite blind, as they nearly all do in time, operation will be necessary, but should only be performed after a thorough examination under atropia. Tenotomy alone rarely suffices, but must usually be combined with advancement of the opposing muscle.

Passing from the eye to its neighbor the ear, there are one or two points in regard to

ACUTE INFLAMMATION OF THE MIDDLE EAR

that I want to speak of, especially its frequency and its importance in children. Many a child is treated for some obscure acute febrile illness till the appearance of discharge at the meatus and disappearance of other symptoms tells the tale. I saw this happen last summer to an excellent practitioner, and Dr. Minor, of this city, called attention before the State Medical Society, in 1897, to the occurrence of this complication in acute illness, being mistaken for cerebral complications. McEwen Smith (New York *Medical Journal*, July 21st, 1894) reports that he saw within three years six children with acute middle-ear diseases unrecognized until just before death. In four a diagnosis of "brain fever" had been made, and in two "meningitis." They could all have been saved by proper care at an early period. Hartman of Berlin (Rev. Mensuelle des Maladies de l'enfance, August, 1895) quotes Kassel as finding otitis in 85 cases in autopsies on 108 infants one month old, and in 38 of the 85 pus containing streptococci and pneumococci was present in the middle ear. He adds 47 autopsies of his own, with 37 cases of otitis, 28 of which were bilateral; and 24 of the 37 died of broncho-pneumonia, showing a relation between the organism in the ear and the disease which caused death. The symptoms he puts down are restlessness, placing the hand to the ear, the cry and symptoms of meningitis. Especially in children suffering from acute intestinal disorders, measles, scarlatina, typhoid fever, pneumonia, and influenza should the ears be watched.

If it occurs, while it is very true that most cases get well of themselves, the same is equally true of all other acute diseases, and is no reason why they should be neglected. In every case of this sort we should bear in mind the possibility of a chronic otorrhea, mastoid and cerebral complications, and even death, since these occurrences are not so rare that we can afford to disregard them. In the treatment of this condition, the various "drops," comprising nearly all known substances, and some unknown, are not only, according to my experience, useless, but positively injurious. I have in a few instances seen earache relieved by sweet-oil and laudanum, but never by any other "drops." We must remember that most cases of acute otitis media are catarrhal, only a few being purulent from the first. Lannois (*Annales des Maladies d' oreille*, May, 1896) has found this secretion to be aseptic, yes, even feebly antiseptic, according to Pierce. But if perforation occurs, either by the processes of nature or by the intervention of the surgeon, what chance is there for this to remain a catarrhal inflammation when the canal is full of rancid oil and deposits from various vegetable preparations, tinctures, etc.? I would advise, briefly, a brisk cathartic, an opiate, and heat, preferably dry heat, continuously applied to the region of the ear, and if the pain is very severe, four leeches in front of the tragus. At this stage Politzerization may be gently done, but usually is best omitted, as it is painful and may drive septic material up from the pharynx. If drops must be used, 15 per cent. carbolic acid in glycerin is the least injurious, though syringing the ear with hot water (105° to 110° F.) will do more good than any other local application. A quart of water at this temperature run into the ear from a fountain syringe every two or three hours, with dry heat applied in the interval, is more important than any other item of the treatment, and should always be used if dry heat does not give prompt relief. If at the end of the twenty-four hours, in spite of this treatment, and especially in spite of the syringing with hot water, the symptoms have not abated, the drum should be incised along the lower and posterior border to let the exuded fluid escape. This should be done with a clean knife, and the canal thoroughly cleansed with a bichloride solution. When the opening is made the fluid, usually bloody serum, may be taken up with sterile cotton swabs, and then a strip of iodoform gauze passed to the depths of the canal, and the hollow of the auricle filled with sterile cotton, held in by collodion applied at several points. The dressing may be changed daily, and the ear syringed gently with a warm bichloride solution.

If the case is left to nature and the drum perforated, the same attention to cleanliness, with insufflation of a small quantity of an impalpable powder of boric acid, will, in the large majority of cases, effect a cure. If neglected they become

CHRONIC OTORRHEA

or "running ears," which are quite common, and the conclusions I have formed about this condition are probably those at which all arrive, and which do not need repetition.

A complication which may arise from the acute or chronic middle ear suppurations, especially the chronic form, is

MASTOID DISEASE

or inflammation of the lining of the cavities of the mastoid process, a condition which has always seemed to me to present many points of analogy to appendicitis. If this is agreed to, then I think I need hardly add that, while spontaneous cure is sometimes seen, the best interest of the patient is often consulted by early radical operation. I do not mean to operate on every case of mastoid disease as soon as the diagnosis is made. The patient should be put to bed, closely watched and treated with local heat or cold, according to the idiosyncrasies of the patient, the stage of the case and the predilection of the attendant, sedatives and attention to the ear trouble. If the mastoid symptoms do not subside promptly, then I believe the operation, of freely opening the mastoid cells, is indicated.

I have done my first and last Wilde's incision, that is, an incision through the skin over the mastoid process, and it is as unsurgical as scarification of the abdomen would be in appendicitis. If the operation for mastoid abscess is attended by some danger, it is infinitely less than that which attends an abscess touching almost vital structure in every direction.

I am told that Politzer, the fatner of modern otology, begins his course of lectures by showing a temporal bone, and remarking that this bone contains the organ of hearing. It is bounded by four sides. One of these sides is life, for by it we communicate with the outside world. The three other sides are death, for through these may disease from the ear attack the brain itself, one of its venous sinuses, or the great vessels of the neck. Surely an abscess situated in such a locality admits of very little discussion as to the wisest means of dealing with it.

I know cases go to a point where pus formation has occurred or *seems to have,* and recover by absorption (?) or spontaneous evacuation, but these deserve to be classed as "anomalies and curiosities of medicine," and should, I think, have little weight in determining the conduct of a case of mastoid inflammation.

[DISCUSSION.

*Dr. J. L. Minor:* I have nothing to add to Dr. Ellett's remarks on ophthalmia neonatorum. In reference to refraction cases he finds it necessary to use a mydriatic. I only use it where there is a spasm of accommodation. In selecting a mydriatic I give the preference to atropine, as I do not think homatropine acts so thoroughly.

As to the management of cases of strabismus, it is hard to make a rule and harder to apply one when made. Generally, though, the use of atropia is an index to the value of glasses. If glasses cannot relieve, an operation is indicated, regardless of the age. I indorse the doctor's treatment of the middie-ear affection, except as to drops. I use a mixture of one part chloroform to seven of olive-oil, and find that dropping this into the ear lessens pain and prevents suppuration. I indorse also his treatment of mastoiditis, with one exception—I commend Wilde's incision. When the disease is subperiostial, a periostial incision will allow the pus to escape and give relief. I recall four cases in which Wilde's incision was all that was necessary. When the disease is in the antrum Wilde's incision may become merely a preparatory step to trephining.

*Dr. A. G. Sinclair:* I approve of Dr. Ellett's paper with one or two exceptions. I find that in very many instances glasses may be fitted without the use of the mydriatic. Where there is any spasm of accommodation it is of course necessary to use one, and I prefer atropine. I approve of Wilde's incision.

*Dr. J. F. Hill:* I indorse the treatment of ophthalmia neonatorum with nitrate of silver and boracic acid. As to the use of mydriatics, I agree with Drs. Sinclair and Minor. In many cases glasses can be fitted perfectly without the aid of a mydriatic. I, too, give atropine the preference.

In the treatment of otitis media I indorse part of Dr. Ellett's paper and part of what the others have said. I do not use drops, nor have I been able to relieve earache with hot water. I prefer dry heat. I consider the use of peroxide of hydrogen after suppuration a dangerous practice, as it sets up a further inflammation. I carefully wipe out the ear with a pledget of cotton, then blow through the Eustachian tube with the Politzer bag.

As to mastoiditis, I believe in making an incision right down through the periosteum, and have been successful with this treatment.

*Dr. T. E. Edwards:* I have for fifteen years tried to keep from getting into habits. I do not use a mydriatic with an intelligent person. I recently had a case of refraction in a lady fifty-two years old. She had had her eyes examined repeatedly, once by Dr. Savage, whom I consider the peer of any oculist, mydriatics having been used several times. Without a mydriatic I fitted her in twenty minutes with glasses which suited perfectly.

As to middle-ear trouble, my plan has been invariably hot fomentation. I always look for pus to be formed, if it is not already present, and pus cannot be absorbed with dry heat. I pour the ear full of hot water and let it remain four or five minutes. I incise when necessary, without injuring the hearing.

In ophthalmia neonatorum I use nitrate of silver. I never use boracic acid, as I do not consider it an antiseptic.

*Dr. Heber Jones:* About the only disease discussed in Dr. Ellett's paper with which a general practitioner has anything to do is ophthalmia neonatorum. In obstetrical practice I have for years made it a custom to wipe off the child's face as soon it is born, and by taking this precaution have had very few cases of this disease. The child does not open its eyes the second it touches the bed, so I just wipe and sometimes wash off the face before it does open up.

*Dr. M. F. Rogers, of New Albany, Miss.:* I always carry a bottle of bichloride tablets with me, and when I haven't the silver nitrate I use the corrosive sublimate, and find that it does just as well. In earache I simply drop a four per cent. solution of cocain into the ear, and it always gives immediate relief.

*Dr. Ellett (closing):* A good deal has been said about the use of mydriatics only in spasm of accommodation. I would like to know how a diagnosis of moderate spasm of accommodation can be made without paralyzing the ciliary muscle. You all know who Dr. Knapp is. Well, one of these gentlemen has told me of seeing a case in which Dr. Knapp had failed to recognize a spasm of accommodation, and gave myopic glasses for a hyperopic eye; and if men of Dr. Knapp's skill make these mistakes, we are all likely to do so unless we guard against it by paralyzing the accommodation.

One of the gentlemen mentioned his admiration for Dr. Savage. Then why don't he follow Dr. Savage's teachings in regard to using mydriatics? Doubt has been expressed as to the efficacy of homatropine, but I am sure this arises from its improper use. If the method I spoke of is adopted, of using the homatropine and cocain disks, three in each eye, with half-hour intervals, I know the cycloplegia is complete at the end of that time. This is the method of Dr. Casey Wood, and in September I saw him in Chicago, and on this subject he told me that he and his assistants had gone over this matter many times, comparing the results with those obtained under atropine, and the effect of the homatropine disks leaves nothing to be desired. Then a matter that was not touched on in the discussion is the "shadow test." I repeat that this is the only objective test of any value, and it is impossible with an undilated pupil. One of the gentleman spoke of testing a woman of fifty-two without a mydriatic. Of course he did. Nobody wants to use a mydriatic in a woman of fifty-two. She has no accommodation to paralyze.

The assertion that boracic acid is not an antiseptic, is not in accordance with the investigations. I think it is used in this disease largely for its mechanical effects in washing away the pus. As regards the chloroform and olive-oil mixture in earache, I know it is highly commended, but I can only say that I have used and have never been able to relieve the pain with it. Cocain has no effect on skin surfaces, such as the drum membrane is, and I am inclined to think the effect following its use is a coincidence.

Now, gentlemen, in regard to Wilde's incision. I submitted a synopsis of my paper to those who were down to open the discussion, and they told me they "came here loaded." Well, I have returned the compliment, and have come here loaded, too. You know that the cavity in the mastoid and the middle ear cavity are practically one, and the mastoid inflammation that comes from middle ear inflammation is, in nineteen out of twenty cases, within the bone, an osteomyelitis, and not a periostitis. Now when it becomes necessary to operate for this condition, I believe the Wilde incision to the bone is useless, and if any operation is necessary it is that of freely opening the bone and attacking the seat of trouble. In support of this view I beg to submit the following:

B. A. Randall, Professor of Otology in the University of Pennsylvania ("The Inefficacy of Wilde's Incision in Mastoid Empyema," *University Medical Magazine*, October, 1897): "Any assumption that real suppuration, still more caries, has been present in or on the mastoid and has been dissipated by treatment is negatived by the actual experience of this and every other branch of surgery. It will require more than X-rays to demonstrate the presence of the caries or the pus which he imagines is there; although it must be freely conceded that the symptoms are strongly suggestive of such conditions. These are the cases for which the Wilde incision and other measures have been employed and urged as sovereign remedies; but clinical experience wholly fails to substantiate the claims of usual or great value. If pus be really present on the surface of the mastoid there is not one chance in twenty that it was formed there. In almost all cases it has burrowed out from the attic or antrum along the canal, or has found its way through the fistulous or cribriform mastoid cortex from the cells within. So it may be laid down as a rule that pus on or in the mastoid is the only indication for the Wilde incision. In the nineteen out of twenty instances where this is the case, as before stated, the pus comes from beyond the mastoid surface; and the section of the soft parts is but a step towards the evacuation and cure. If the knife be really needed, the chisel or spoon is required."

Chipault and Demoulin, *Annals of Ophthalmology and Otology*, October, 1895: "Wilde's incision is rarely indicated. In the majority of cases its employment is not reasonable because it does not attack the seat of the trouble, but merely relieves a symptom—pain."

Broca and Lubet (*Rev. des Sciences Med. en France et a l'étranger*, July 15, 1895) strongly denounce Wilde's incision, and believe it should be abandoned for more radical measures.

Howard Lilienthal, a surgeon of no mean repute (*N. Y. Med. Journal*, June 1, 1895): "I have yet to learn a satisfactory reason for delay where there has recently been middle ear suppuration and where marked mastoid tenderness exists. The stake is a heavy one, being nothing less than a human life; the risk of operation is a comparatively slight one."

Frank S. Milbury (*Med. Record*, November 13, 1897): "This is practically what is known as Wilde's incision. In the adult experience has taught all operators that it is not advisable to stop here, as the cortex is too dense and non-permeable. It certainly is not wise to do this and delay to ascertain what further may develop, and subject the patient to a second anesthetization and operation, when the whole work should have been completed at first."

My experience with these and many other similar published opinions are the reasons why I oppose Wilde's incision.]

